# The Impact of the COVID‐19 Pandemic on Pattern of Antibiotic and Opioid Prescriptions by Dentists in Alberta, Canada

**DOI:** 10.1002/cre2.913

**Published:** 2024-07-07

**Authors:** Riley Immel, Babak Bohlouli, Maryam Amin

**Affiliations:** ^1^ School of Dentistry University of Alberta Edmonton Alberta Canada

**Keywords:** antibiotics, COVID‐19 pandemic, electronic health records, opioids

## Abstract

**Objectives:**

After the shutdown of most dental services during the COVID‐19 lockdown, the oral health community was concerned about an increase in prescribing opioids and antibiotics by dentists due to patients' limited access to dental offices. Therefore, the objective of this study was to investigate the impact of COVID‐19 pandemic on the pattern of antibiotic and opioid prescriptions by dentists in Alberta, Canada.

**Methods:**

Data obtained from the Tracked Prescription Program were divided into antibiotics and opioids. Time periods were outlined as pre‐, during‐, and postlockdown (phase 1 and 2). For the number of prescriptions and average supply, each monthly average was compared to the corresponding prelockdown monthly average, using descriptive analysis. Time series analyses were conducted using regression analyses with an autoregressive error model. Data were trained and tested on monthly observations before lockdown and predicted for during‐ and postlockdown.

**Results:**

A total of 1.1 million antibiotics and 400,000 opioids dispense were tracked. Decreases in the number of prescriptions during lockdown presented for antibiotics (*n* = 24,933 vs. 18,884) and opioids (*n* = 8892 vs. 6051). Average supplies (days) for the antibiotics (*n* = 7.10 vs. 7.55) and opioids (*n* = 3.92 vs. 4.05) were higher during the lockdown period. In the trend analyses, the monthly number of antibiotic and opioid prescriptions showed the same pattern and decreased during lockdown.

**Conclusion:**

The COVID‐19 pandemic altered the trends of prescribing antibiotics and opioids by dentists. The full impact of COVID‐19 pandemic on the population's oral health in light of changes in prescribing practices by dentists during and after lockdown warrants further investigation.

## 1 Introduction

Background rationale: While much attention was directed toward the broader health implications of the COVID‐19 pandemic, the impact on oral health received less consideration. In Alberta, Canada, the Alberta Dental Association and College (ADA&C) imposed restrictions on nonemergency dental procedures from March 17 to May 14, 2020, resulting in a significant decrease in dental services provided in the public dental health clinics in Alberta because of the pandemic (Rabie and Figueiredo 2021). During the first wave (March 17–October 31, 2020), these clinics treated 46% fewer patients compared to the previous year, with a notable increase in dental prescriptions. Both in‐person and teledentistry consultations were utilized for patient care. Antibiotic and opioid prescriptions from these clinics surged by 76% and 44%, respectively, during this period (Rabie and Figueiredo 2021). Nevertheless, questions persist regarding the typical prescribing practices of nonpublic health dentists before the pandemic, during the lockdown, and the post‐COVID effects.

Before the COVID‐19 pandemic, Suda et al. (2019) reported that out of all prescriptions written by United States dentists, 22.3% were opioids. Hydrocodone‐containing opioids were the largest proportion of opioid drugs prescribed by US dentists at 62.3%. Codeine followed hydrocodone at 23.2% of all opioids prescribed by US dentists, and oxycodone and tramadol made up 9.1% and 4.8%, respectively (Suda et al. 2019). Falk et al. (2019) reported that out of all the opioid prescriptions written by Manitoba dentists during 2014–2017, 97.4% contained codeine, 1.7% contained tramadol, and 0.7% contained oxycodone. Including other healthcare practitioners, Manitoba dentists wrote 3.8% of all opioid prescriptions in the province (Falk et al. 2019). However, it should be noted that opioid prescriptions by dentists have decreased steadily in recent years, at least in pediatric populations (Matthews et al. 2019). Antibiotic prescriptions in the United States by general dentists before the COVID‐19 pandemic included a majority of Amoxicillin (56.3%), followed by Clindamycin (14.4%), Penicillin V (13.2%), Cephalexin (4.9%), and Azithromycin (4.7%) (Roberts et al. 2017).

In England, researchers found a 25% increase in the amount of dental prescriptions for antibiotics during April–July 2020 in comparison to 2019 (Shah, Wordley, and Thompson 2020). In Australia, there were 16% and 18% decreases in antibiotic and opioid prescribing during April 2020 in comparison to previous years (Mian, Teoh, and Hopcraft 2021).

Despite the decline in opioid prescriptions by dentists observed in certain regions, global concerns persist regarding opioid usage. Furthermore, there is notable diversity in antibiotic prescribing practices among dentists worldwide, with distinct trends discernible across various countries. The COVID‐19 pandemic has introduced further complexities, with some regions experiencing increases in antibiotic prescriptions. Understanding the patterns and trends of opioid and antibiotic prescribing by dentists pre‐ and post‐pandemic is essential for the effective management of pain and infection in dental practice.

## 2 Objectives

The aims of this study were to analyze the prescribing trends of dentists in Alberta, as well as to examine the number of prescriptions and average supply (in days) of medications on a month‐to‐month basis to the prelockdown, during lockdown, and postlockdown periods. Based on previous similar studies, it was hypothesized that there would be an increase in both the number of prescriptions and average supply (days) for each dispensation when compared with previous years.

## 3 Methods

### 3.1 Study Design

Ethics approval was obtained from the University of Alberta Research Ethics Board (Protocol Numbers: Pro00095759_AME3 and Pro00105754). This study uses time series analysis along with regression analysis and an autoregressive error model to analyze the impact of COVID‐19 on dental prescribing trends.

### 3.2 Participants

The project used a retrospective population‐based cohort of patients treated by selective classes of drugs. The patients included were adults and children who received a prescription for antibiotics or opioids by Alberta dentists during the COVID‐19 pandemic from 2020 to 2021. Only prescriptions filled at a community pharmacy were included in the study. These data were then compared with prescriptions of the same drug classes written by Alberta dentists in 2018 and 2019.

### 3.3 Setting/Variables/Data Sources/Study Size/Data Access and Cleaning Methods

Detailed patient data including their age, gender, medications, provider, and duration of medication prescribed were obtained from the College of Physicians and Surgeons of Alberta (CPSA) as part of the Tracked Prescription Program (TPP) to align with the years (2018–2021) and provider (dentist) of interest. TPP uses data from the Pharmaceutical Information Network (PIN) to extract medications using Drug Identification Numbers (DIN) from dispense records belonging to community pharmacies in Alberta.

### 3.4 Quantitative Variables

Individual drugs were grouped into antibiotics or opioids, following the classifications of the TPP. Opioids were further narrowed to only include the top 5 prescribed as it was 99.9% of all prescriptions written by Alberta dentists for opioids and other painkiller drugs. A monthly number of prescriptions and average supply of medication for total prescriptions, each drug classification, and individual drugs from January 2018 to December 2021 were obtained.

### 3.5 Statistical Methods/Bias

The monthly average comparison of prelockdown, during lockdown, and postlockdown was conducted for descriptive analysis comparison. To avoid the seasonality impact on the analyses, the monthly average of March–May in 2018 and 2019, collectively, was considered prelockdown period, and the average of March–May 2020 was defined as during lockdown. Two postlockdown periods were defined as phase 1 (June–December 2020) and phase 2 (June–December 2021) and separately compared with the monthly average of the same months in 2018 and 2019. The lockdown period mirrors the restrictions against nonemergent treatment set in place on March 17, 2020, by the Alberta Dental Association and College and lifted on May 14, 2020 (ADA&C 2021). For the number of prescriptions and average supply of medication, the monthly average during the lockdown and post‐ockdown periods were compared to the monthly average of the same period in prelockdown. To describe demographics, categorical variables were reported with percentages and continuous variables with means, standard deviations, and ranges, as appropriate.

Changes in the number of prescriptions for antibiotics and opioids were investigated using time series analysis. Time series analysis was conducted to examine if the trend over time was affected by COVID‐19 during the lockdown period. We utilized regression analyses with an autoregressive error model to address population changes. Data from previous timestamps were analyzed to forecast trends, comparing predicted and actual numbers across subsequent timestamps using confidence intervals. In our study, training data spanned the prelockdown period with 26 timestamps, enabling the interpretation of variations between predicted and actual numbers. Linear trends and seasonal variables were added to the model. Using the month of the year, 11 dummy variables for the month of the year were created. To avoid multicollinearity, of 12 months, we created 11 dummy variables, and the absent variable was considered baseline. The prediction model was conducted during the lockdown period to determine the expected versus the observed number of dispenses. A 95% confidence interval (CI) around expected numbers was defined to determine significant level changes in observed values. Those observed numbers that fall within the 95% CI of expectations were considered a nonsignificant change and any observed numbers out of the 95% CI were reported as a significant change. In the figures, 95% CI was presented as lower and upper limits of the expected numbers along with observed numbers of monthly prescriptions. Analysis was conducted using the SAS enterprise guide.

## 4 Results

### 4.1 Participants/Descriptive Data

Within the study period, approximately 1.1 million antibiotic and 400,000 opioid prescriptions were distributed. The five main antibiotics prescribed by dentists in Alberta were amoxicillin (70.2%), clindamycin (11.0%), penicillin (7.3%), metronidazole (3.5%), and amoxicillin‐clavulanic acid (3.4%) and the five main opioids were codeine (88.5%), tramadol (9.1%), oxycodone (1.5%), morphine (0.6%), and hydromorphone (0.1%).

Females received 51.8% of antibiotic prescriptions and 49.7% of opioid prescriptions, whereas males received 48.2% and 50.3%, respectively. The mean (SD) age of females and males receiving antibiotic prescriptions was 46.7 (20.1) and 47.8 (20.0) years, respectively. For opioid prescriptions, the mean (SD) age of females and males were 39.8 (17.8) and 42.5 (17.9) years, respectively. Antibiotics were prescribed in the highest proportion to the 41–65 years age category (42.6%). The number of opioid prescriptions was highest for the 19–40 years age category (41.9%) (Table 1).

**Table 1:** Number of prescriptions, mean age (SD), as well as sex and various age groupings for antibiotic and opioid prescriptions, 2018–2021.

	Number of antibiotic prescriptions (%)	Mean age (SD) of prescription for antibiotics	Number of opioid prescriptions (%)	Mean age (SD) of prescription for opioids
Sex	
Male	562,538 (48.2%)	47.8 (20.0)	204,414 (50.3%)	42.5 (17.9)
Female	605,407 (51.8%)	46.7 (20.1)	202,143 (49.7%)	39.8 (17.8)
Total (%)	1,167,945 (100.0%)	‐	406,557 (100.0%)	‐
Age (years)				
0–18	110,107 (9.4%)	12.6 (4.9)	41,254 (10.1%)	16.6 (1.7)
19–40	328,160 (28.1%)	30.0 (6.4)	170,308 (41.9%)	28.9 (6.5)
41–65	496,979 (42.6%)	53.9 (7.1)	153,613 (37.8%)	52.7 (7.1)
66–105	232,699 (19.9%)	74.0 (6.7)	41,382 (10.2%)	73.0 (6.3)
Total (%)	1,167,945 (100.0%)	‐	406,557 (100.0%)	‐

*Note*: Table representing demographic data of the population studied.

During the lockdown period, as compared to the prelockdown, the absolute monthly average number of prescriptions of both antibiotics (*n* = 24,933 vs. 18,884) and opioids (*n* = 8892 vs. 6051) decreased 32% and 47%, respectively. In phase 1 postlockdown period, the average monthly number of antibiotic prescriptions was 7% higher than the prelockdown period (*n* = 25,124 vs. 23,586). In phase 2 postlockdown, however, there was a 9% increase in number of prescriptions compared to the same time period in the prelockdown (*n* = 23,586 vs. 25,791). Monthly average number of opioid prescriptions in phase 1 (*n* = 8860 vs. 8418) and phase 2 postlockdown (*n* = 8587 vs. 8418) increased 5% and 2%, respectively, as compared to the same time period in the prelockdown (Table 2 and Appendix S1).

**Table 2:** Average monthly number of prescriptions/days supply for antibiotics and opioids.

	Periods		
Month	Average of 2018 and 2019	Average 2020	Average 2021
Monthly prescription			
Antibiotic			
March–May	24,933	18,884	
June–December	23,586	25,124	25,791
Opioids			
March–May	8892	6051	
June–December	8418	8860	8587
Monthly average supply (days)			
Antibiotic			
March–May	7.1	7.55	
June–December	7.13	7.17	7.12
Opioids			
March–May	3.92	4.05	
June–December	3.9	3.91	3.86

*Note*: Demographic data for the average monthly number/days supply for antibiotics and opioids.

In the lockdown period, there were 6% and 3% increases in the average supply (days) of prescribed antibiotics (*n* = 7.10 vs. 7.55) and opioids (*n* = 3.92 vs. 4.05) (Table 2). While in phase 1 postlockdown period, an increase was observed in the average supply (days) of antibiotics (*n* = 7.13 vs. 7.17), there was almost no change of that in phase 2 (*n* = 7.13 vs. 7.12) (Table 2). While monthly average supply (days) for the opioid prescriptions was stable with almost no change in phase 1 postlockdown (*n* = 3.90 vs. 3.91), the average days of supply decreased in phase 2 postlockdown (*n* = 3.90 vs. 3.86) (Table 2).

### 4.2 Outcome Data/Main Results

**Figure 1:**
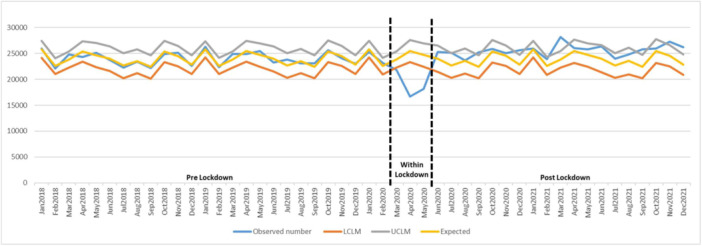
Antibiotic dispenses trend from Jan 2018 to Dec 2021, including upper limit of model (UCLM), lower limit of model (LCLM), expected, and observed numbers. Number of antibiotic prescriptions from 2018 to 2021 including predicted upper and lower limits (expected) and observed numbers.

**Figure 2:**
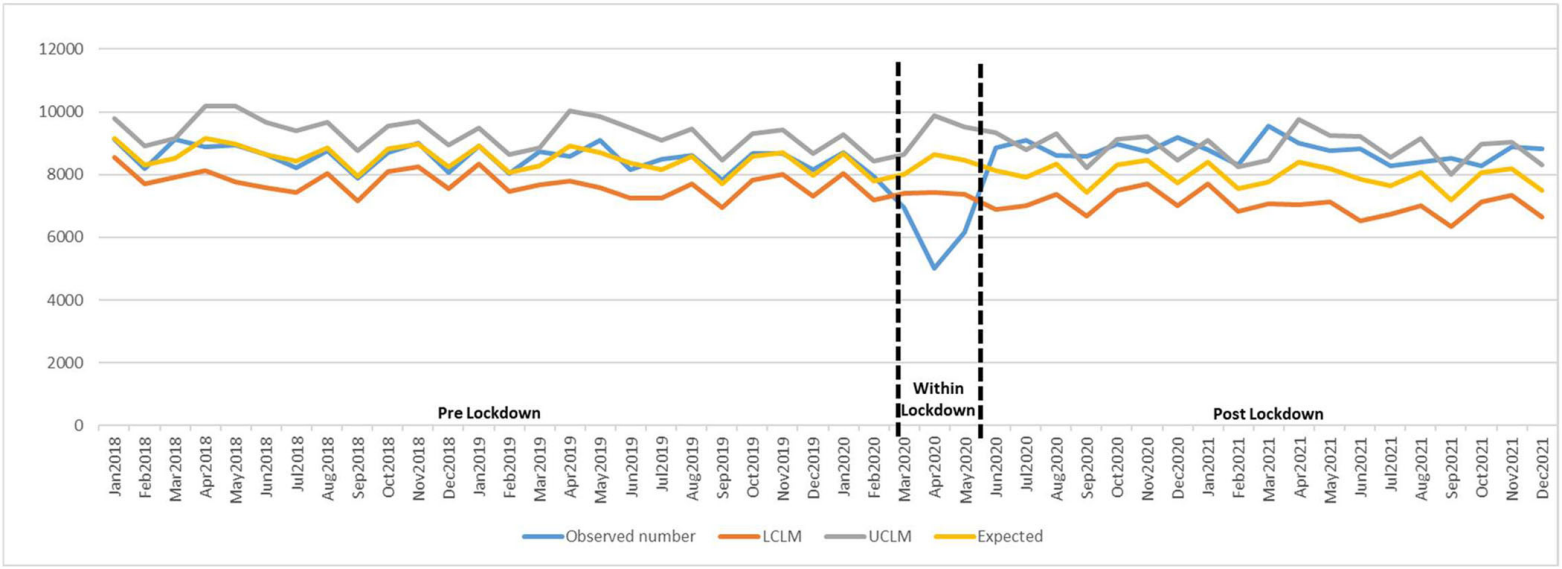
Opioid dispenses trend from Jan 2018 to Dec 2021, including upper limit of model (UCLM), lower limit of model (LCLM), expected, and observed number. Number of opioid prescriptions from 2018 to 2021 including predicted upper and lower limits (expected) and observed numbers.

In the trend analyses, the monthly number of antibiotic and opioid prescriptions showed the same pattern and decreased during the lockdown. The observed number of prescriptions for both medications was out of the lower 95% CI for the expected numbers, indicating statistically significant changes (Figures 1 and 2). An upward trend was noticed starting postlockdown and the observed number of dispensations was higher than expected numbers for the rest of the study period. Figure 1 presents that the number of antibiotic dispensations after the lockdown period remained over predicted numbers; however, at some points, there was a statistically significant increase in the prescription numbers. For opioid dispenses, an upward trend started in June 2020 and remained over the expected numbers to the end of the study; however, in some periods, statistically significant changes were noticed (Figure 2).

## 5 Discussion

### 5.1 Key Results

Overall, the COVID‐19 pandemic had profound effects on prescribing trends in dentistry, in the areas of the total number of prescriptions and the average supply of medication prescribed for antibiotics and opioids. The effect was particularly evident during the lockdown months of March–May 2020, with large decreases in the number of prescriptions for both drug categories. Following the lockdown period, marked increases were seen in the number of prescriptions for most of the months in both drug classes. Increases on average supply (days) were also noted for antibiotics and opioids during the lockdown period and were relatively consistent in the following months. Antibiotics were most prescribed to individuals aged 41–65 years, whereas opioids were most frequently prescribed to those aged 19–40 years.

### 5.2 Interpretation

To some oral healthcare professionals, dental services were not given high enough priority by World Health Organization officials during their recommendation to cease all nonessential dental services in favor of remote options (WHO 2020). These nonessential dental services include: “oral health check ups, dental cleanings, and [other] preventative care” (WHO 2020). The American Dental Association disagreed with the WHO's recommendations, publishing a new interim policy with the title: “Dentistry is Essential Health Care (COVID‐19)” (American Dental Association 2020). In this new interim policy, they sought to redefine Essential Dental Care to include preventative measures, and to update the existing terms Emergency and Elective Dental Care in use by media, the WHO, policy makers, and others, with the term Essential Dental Care (American Dental Association 2020).

The long‐term effects of this shutdown of “nonessential” dental care are widespread but were especially significant with regard to analgesic and antibiotic prescriptions to patients. During the pandemic, the WHO requested Oral Health Care professionals to conduct emergency dental care remotely wherever possible using the 3 A's: advice, analgesics, and antibiotics (WHO 2020). Although these were taken as emergency measures, without being able to take radiographs or conduct proper intra and extraoral examinations, some might wonder if these drugs were being prescribed in under normal circumstances. However, prescriptions written by dentists are only one part of delivering oral health care and do not fully represent whether the oral health care needs of patients were being adequately taken care of during the COVID‐19 pandemic.

In our study, amoxicillin was the most frequently prescribed antibiotic overall, followed by clindamycin. Prescriptions for antibiotics were similar to findings in the United States, with the same three (amoxicillin, clindamycin, and penicillin) being the most prescribed (Roberts et al. 2017). Opioid prescribing trends by dentists in Alberta were also similar to Manitoba where codeine and tramadol made up the majority of prescriptions (Falk et al. 2019). In comparison to US dentists, the results were markedly different where hydrocodone‐containing opioids were mainly used (Suda et al. 2019).

This study had similar results to the dental prescribing study in Australia, with decreases in antibiotic and opioid (codeine and tramadol) prescriptions during the April 2020 (Mian, Teoh, and Hopcraft 2021) lockdown. This is likely a result of the nearly identical measures that the Australian Health Protection Plan Committee enacted in comparison to Alberta: emergency treatment only and the deferral of all routine dental care. Another factor to consider is the possibility of patients seeking relief from dental pain or infection in Hospital Emergency Departments and other acute care facilities. This could potentially influence dentists' prescription rates during the COVID‐19 shutdown. However, these results contrasted with the dental prescribing study in England, where increases in antibiotic prescription during April 2020 were found, but were similar in that antibiotic prescription was also significantly increased in June and July 2020 in Alberta (Shah, Wordley, and Thompson 2020). England implemented the same deferral of nonurgent care and limited treatments to emergency only. A possible reason to explain the difference is that the UK National Health Service directly enacted the principles of AAA—advice, analgesics, and antibiotics (Hurley and Neligan 2020). While the WHO endorsed the AAA principle, the Alberta Dental Association and College (ADA&C) did not explicitly cite this recommendation. Instead, in their “COVID‐19 Emergency Dental Protocol”, the ADA&C outlined specific criteria for dental emergencies. According to their protocol, conditions such as orofacial trauma, significant infection, prolonged bleeding, or severe pain, unmanageable through alternative therapies like teledentistry or pharmacotherapy, warrant an in‐person assessment (ADA&C 2021).

However, the 2022 study by Thompson et al. highlighted significant variations in dental prescribing practices in the United Kingdom, United States, Australia, and Canada. Given this diversity, it becomes challenging to speculate on the reasons behind differing results pre‐, intra‐, and post‐COVID, as numerous factors influencing dental prescribing practices differ across these countries. For instance, England's guidelines typically do not advocate for antibiotic prophylaxis to prevent infective endocarditis following a dental procedure, unlike the other three countries. Consequently, it is anticipated that England's prescription rates would have experienced a greater increase from baseline during lockdown, as their prescribing practices do not adjust for this decrease as observed in the other three countries (Thompson et al. 2022).

This study expands on the previous studies conducted in the United Kingdom and Australia as it involves postlockdown results. It also maximizes the accuracy of prelockdown data by using monthly averages from 2018 to 2019 to avoid increases in prescriptions being attributable to increases in population as much as possible. This is the first study with this large of a timeline. It aligns with other studies in the medical field that also found increases in the usage of certain medications (such as opioids) postlockdown (Campitelli et al. 2021).

### 5.3 Generalizability

The reduced antibiotic prescriptions in the lockdown months of 2020 possibly indicated that serious orofacial infections requiring antibiotics were being undertreated. During the lockdown period in Alberta, dental infections and lesions were found to be increased in comparison to previous years, even though the number of patients was decreased—further suggesting that patients were not having their oral health needs addressed properly (Moharrami, Bohlouli, and Amin 2022). The increase in the average supply (days) of antibiotics found during lockdown also suggests another issue.

The issue with overprescription of longer durations of antibiotics is found in Spellberg's (2016) article, “The New Antibiotic Mantra‐Shorter is Better.” According to Spellberg, antibiotics must be prescribed with the shortest effective duration to minimize outcomes such as the development of antibiotic resistance, and in fact, inappropriate long‐term antibiotic prescriptions have the potential for an increased risk of bacteria that are resistant to antibiotic treatment. Long courses of antibiotics can select for resistant bacteria among our normal microflora (Spellberg 2016). Additionally, evidence of long‐term backlog and the potential worsening of patients' infections could be suggested by the increased prescription of antibiotics in the postlockdown period. A limitation, however, is that the prescription of antibiotics by dentists may not mirror the presence of orofacial infections in patients.

A potential reason for the rise in the average supply (days) of prescribed antibiotics could be dentists' desire to ensure their patients had an ample medication supply for various reasons. These reasons include the potential overwhelming or closure of pharmacies and hospitals, shortages of medication, increased challenges or unpredictability in contacting patients—especially those, such as some seniors, facing technological difficulties with teledentistry for scheduling appointments—and the continually evolving guidelines during the pandemic. Establishing a predefined plan of action for future pandemics would be crucial to mitigate similar issues of confusion and resource scarcity. Moreover, exploring enhancements to teledentistry could prove beneficial for addressing similar situations in the future, provided that accessibility, proficiency in technology use, and comprehension of its functionality are ensured for all users.

Dentists are one of the six source groups that contribute heavily to opioid initiation, or the first time that a patient is exposed to opioids (Pasricha et al. 2018). The misuse of opioids can often occur from the initial exposure to a prescription (Lombardi, Lam, and Ouanounou 2020). There is also a greater chance of developing opioid misuse disorder from longer prescription durations of the drugs (National Institute on Drug Abuse 2022). In the present study, it was found that individuals aged 18–25, who are reported to have the greatest nonmedical use of opioids according to the National Academies of Sciences, Engineering, and Medicine (National Academies of Sciences, Engineering, and Medicine [NASEM] 2017), accounted for 18.6% of all opioid prescriptions within this narrow 7‐year age range. Dentists in Nova Scotia were found to prescribe 60% of all the opioids that children received (Matthews et al. 2019). These facts combine to suggest the creation of a problematic environment from the significant increases in the average supply of opioids by dentists during the lockdown period.

### 5.4 Limitations

A limitation of this study was the use of administrative data, which are raw data not designed for research, and results should therefore be interpreted with caution. Seasonality and significant increases due to population change were considered in the use of time series analysis.

## 6 Conclusion

The number of prescriptions for antibiotics and opioids decreased during the lockdown months, suggesting that patients may have been undertreated for serious infections/pain requiring opioids or antibiotics. During this same period, the average supply (days) of antibiotics and opioids increased, possibly having detrimental effects with regard to antibiotic resistance and opioid initiation in younger patients. Antibiotic prescriptions were increased postlockdown, suggestive of a long‐term backlog of patients caused by the lack of treatment during the lockdown months. More investigation is needed to further understand the impact of modifying dental care during global emergency situations.

## Author Contributions

Riley Immel contributed to cleaning and coding data, data analysis, and interpretation, drafted, and critically revised the manuscript. Babak Bohlouli contributed to conception, study design, data acquisition, statistical analysis, interpretation of results, critically revised manuscript, and gave final approval. Maryam Amin contributed to conception, study design, data acquisition, and interpretation of the results, critically revised the manuscript, and gave final approval. All authors gave their final approval and agreed to be accountable for all aspects of the work.

## Conflicts of Interest

The authors declare no conflicts of interest.

## Accessibility of Protocol and Raw Data

Please contact the corresponding author for information or questions regarding the raw data or protocol used for this study.

## Supporting information

Supporting information.

## Data Availability

The data that support the findings of this study are available on request from the corresponding author. The data are not publicly available due to privacy or ethical restrictions.
