# Supplementary figures and images for: The Impact of the COVID‐19 Pandemic on Pattern of Antibiotic and Opioid Prescriptions by Dentists in Alberta, Canada
Source: Clin Exp Dent Res. 2024 Jul 7;10(4):e913. doi: 10.1002/cre2.913 (PMC11228344; doi:10.1002/cre2.913)

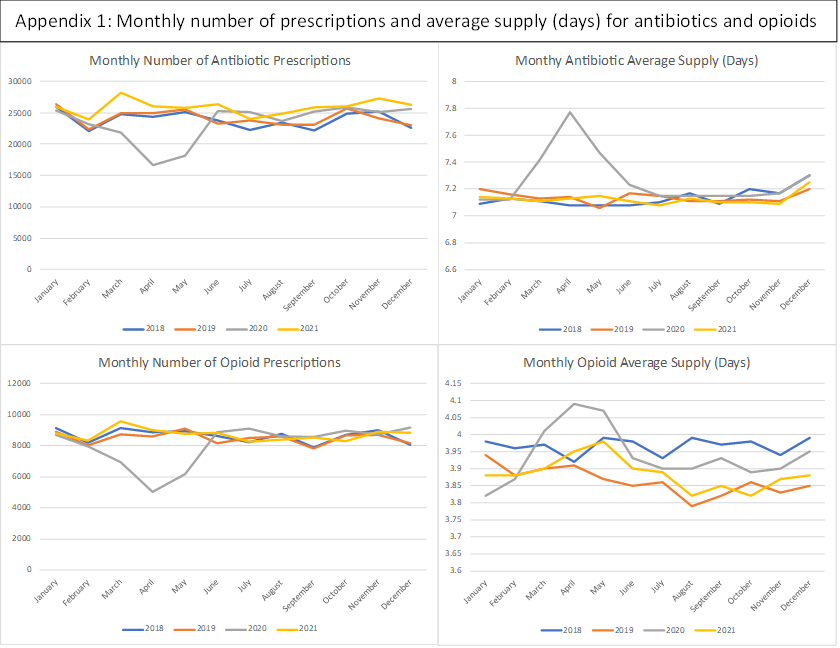

Supplement: Supplementary file 1 — Supporting information. [file CRE2-10-e913-s001.docx]
